# Rotator Cuff Repair Augmented With Interpositional Nanofiber Scaffold

**DOI:** 10.1016/j.eats.2022.08.061

**Published:** 2022-12-21

**Authors:** Casey M. Beleckas, Shariff K. Bishai, Brian L. Badman

**Affiliations:** a Department of Orthopaedic Surgery, Indiana University School of Medicine, Indianapolis, Indiana, U.S.A.; b Associated Orthopedists of Detroit, Saint Clair Shores, Michigan, U.S.A.; c Central Indiana Orthopedics, Fishers, Indiana, U.S.A.

## Abstract

Despite advances in arthroscopic rotator cuff repair, failure rates up to 94% have been reported in the literature for large tears, with rates as high as 36% for small and medium tears. One strategy for improving outcomes is augmentation with a patch, which has typically been incorporated onto the bursal portion of the repaired tendon and been made up of either dermal or bovine collagen tissue. The Rotium wick (Atreon Orthopedics, Columbus, OH)—an interpositional augmentation—is a nanofiber scaffold that is meant to be sandwiched between the rotator cuff and humerus at the bone-tendon interface and is currently the only implant approved by the US Food and Drug Administration to be used in this manner. The scaffold works to improve the cellular organization of the basement membrane during tendon healing at the enthesis and, in a recent sheep study, has been shown to better replicate the natural Sharpey-like fibers similar to the native tendon and increase the strength of the repair more rapidly. The purpose of this Technical Note is to describe the means for use of an interpositional nanofiber scaffold for arthroscopic rotator cuff repair.

With over 17 million Americans experiencing symptomatic rotator cuff injuries, rotator cuff pathology represents one of the most commonly reported musculoskeletal complaints.^1^ For patients in whom conservative management fails, operative rotator cuff repair is frequently performed. Failure of repair is a common complication, more commonly occurring in those with large tears.^2^^,^^3^ However, failure rates of up to 36% have been reported for small to medium size tears as well.^2^^,^^4^

To address the high rates of repair failure, the use of both natural and synthetic scaffolds in conjunction with rotator cuff repair has been implemented. Scaffolds are thought to provide 2 benefits: reducing tension through the repaired tendon during the early healing period by off-loading the tendon-bone construct and providing biological augmentation.^5^^,^^6^ Several such scaffolds that are affixed to the bursal side of the repair have shown reduced failure rates as well as improved clinical and functional outcomes when compared with repair without augmentation.^5^^,^^6^

In 2019, a synthetic nanofiber scaffold (Rotium wick; Atreon Orthopedics, Columbus, OH) was approved by the US Food and Drug Administration for use as an interpositional implant between the tendon and bone in conjunction with rotator cuff repair using suture anchors. This scaffold is both easy to use and theorized to promote healing at the bone-tendon interface. It has shown promising early results in both animal and human studies.^7^^,^^8^ The purpose of this article is to describe the technique for use of this interpositional nanofiber scaffold in the setting of an arthroscopic rotator cuff repair.

## Technique

**Fig 1:**
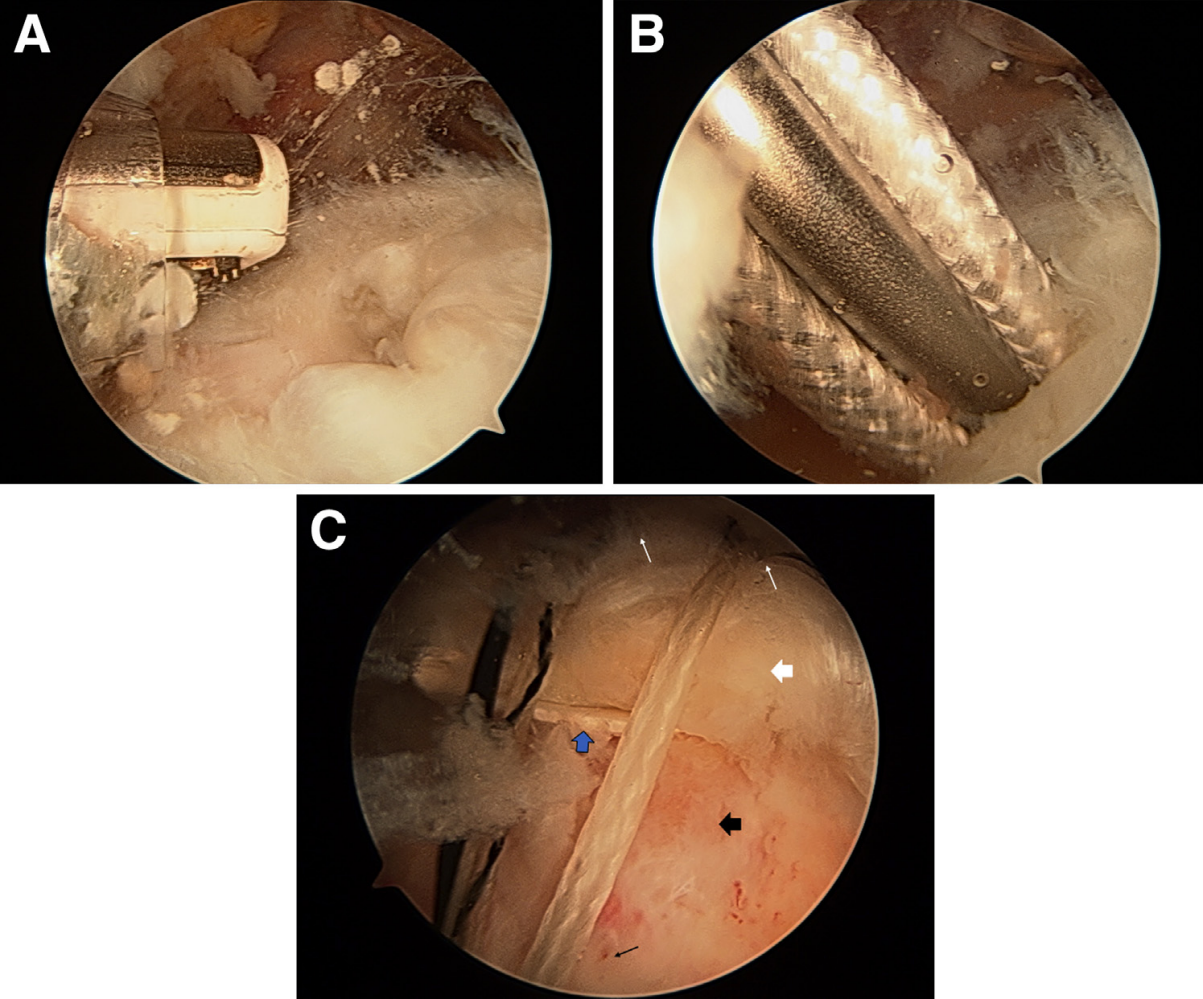
Intraoperative arthroscopic images of a right shoulder taken through the anterolateral viewing portal. (A) A burner is used to clear off soft tissue from the supraspinatus insertion on the humeral head prior to the use of an awl to create marrow vents to induce healing of the rotator cuff repair. (B) A preloaded anchor is inserted into the supraspinatus insertion site. The sutures attached to this anchor will be passed through the center of the nanofiber scaffold. (C) The sutures from the medial row (thin white arrows) pass over the supraspinatus to the lateral row (thin black arrow). The scaffold (thick blue arrow) can be seen sandwiched between the humeral head (thick black arrow) and supraspinatus tendon (thick white arrow).

The procedure is performed with the patient under general anesthesia and positioned for standard arthroscopic rotator cuff repair, whether in the beach-chair or lateral position. Diagnostic arthroscopy is performed with standard evaluation of the tendons, as well as biceps tenodesis or tenotomy and subacromial decompression as indicated, via the surgeon’s preferred portals. The greater tuberosity is then prepared by clearing off the soft tissues (Fig 1A) with a burr or shaver via the lateral portal with the camera in the posterior portal, followed by the creation of marrow vents with an awl to create bleeding bone (Video 1). The anchors (Mitek) are then positioned in the center of the tear with sutures exiting through the lateral cannula (Arthrex) (Fig 1B).

### Implant Insertion

**Fig 2:**
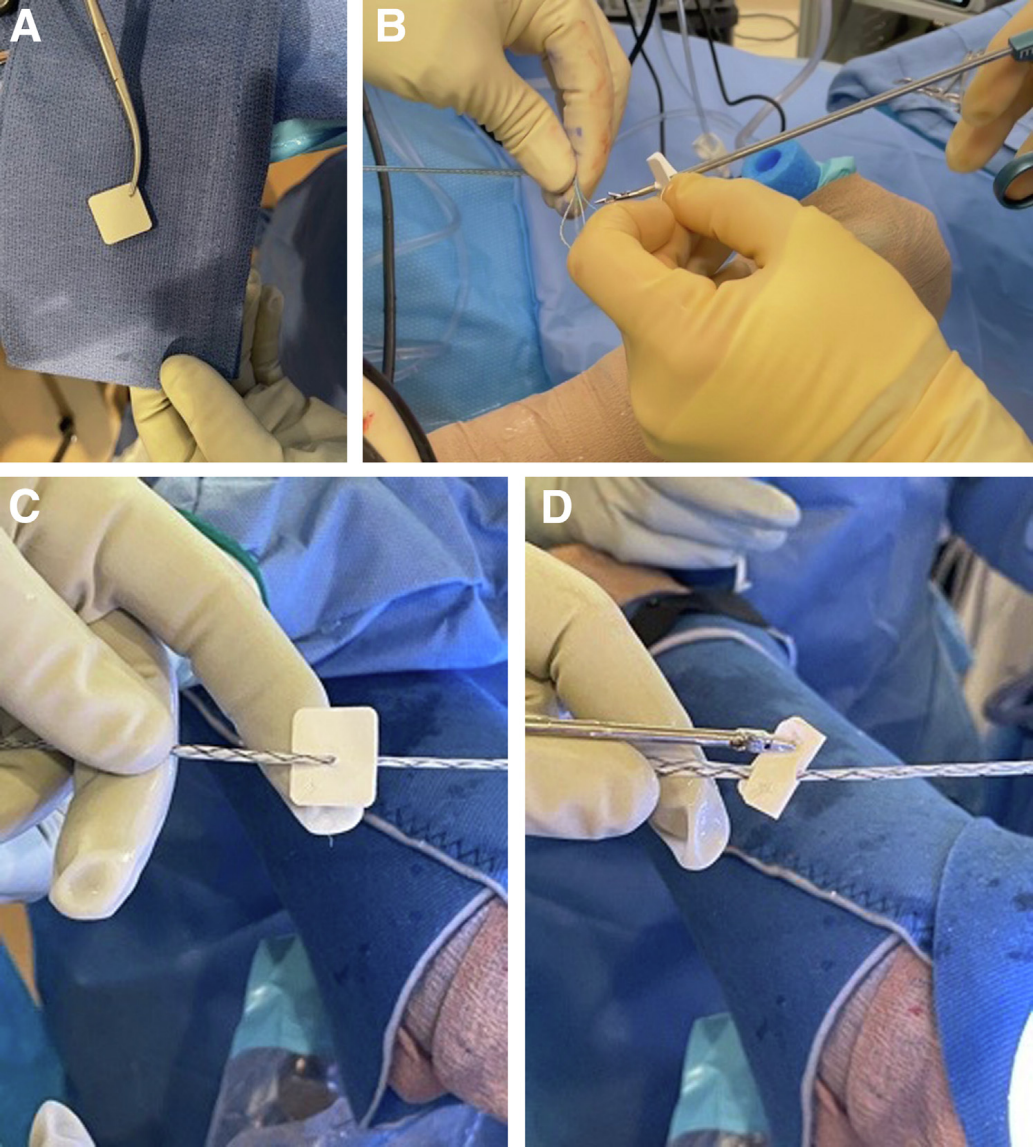
(A) Nanofiber scaffold (Rotium) prior to insertion. (B) A bird-beak grasper is inserted through the central portion of the scaffold, opened to grasp the medial-row anchors, and then closed to pull the sutures back through the center. (C) The scaffold is advanced gently down the medial-row sutures. (D) Prior to insertion through the lateral cannula, the scaffold is either folded in half or gripped in the center near the suture insertion to gently slide down the suture while tension is held on the suture.

**Fig 3:**
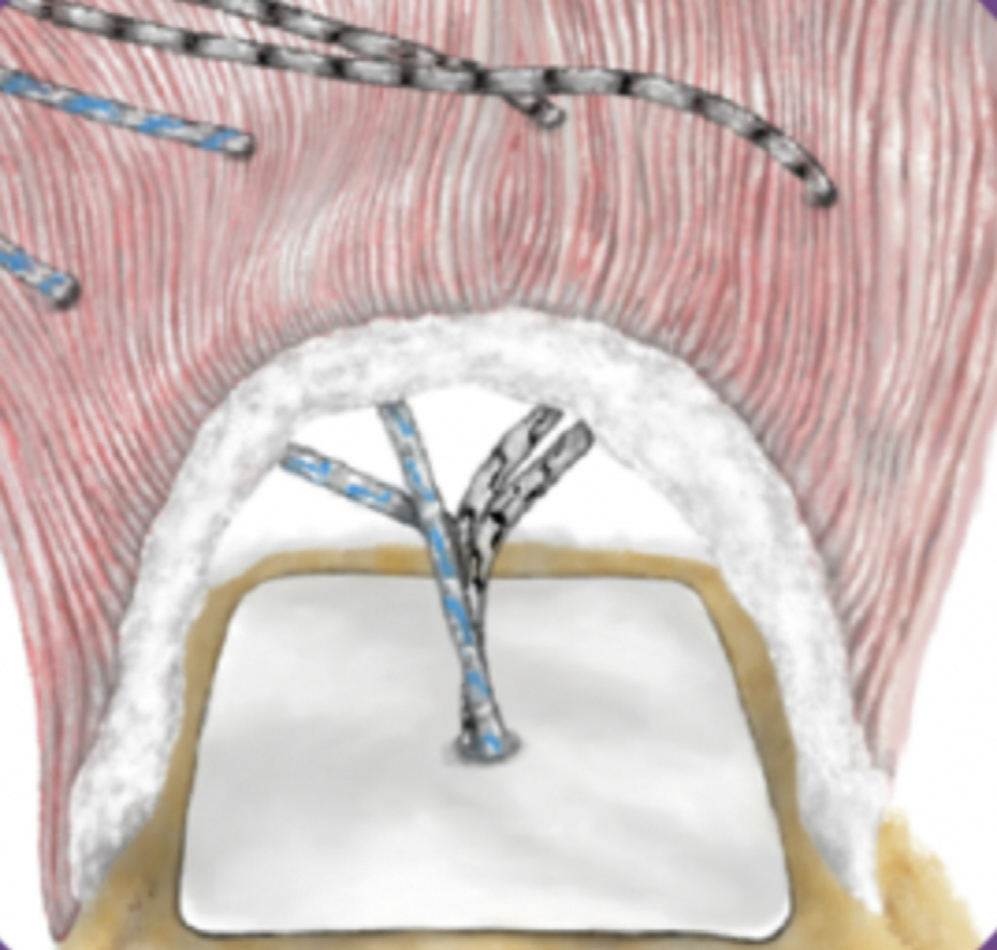
Nanofiber scaffold after placement underneath rotator cuff. The placement of the scaffold between the humeral head and the superior cuff tendons should be noted. Medial-row sutures are then passed through the rotator cuff in standard fashion. (Reprinted from Atreon Orthopedics with permission.)

Once the intended medial-row anchors are placed, the Rotium wick may be passed onto the sterile field (Fig 2A). A bird-beak device (penetrating suture grasper; DePuy Mitek, Raynham, MA) is placed through the center of the wick with the grasper closed (Fig 2B). Sutures from the anchors are grasped with the bird-beak device and pulled through the center of the wick (Fig 2C). With tension on the sutures, the wick can be slid down the sutures toward the anchors through the cannula. This may be performed either by folding the wick in half and pushing with a grasper or by grasping the center of the wick near the sutures and sliding the wick gently through the lateral cannula to the humeral head (Fig 2D). The wick is then positioned onto the prepared greater tuberosity surface and laid flat so as to sit below the articular layer of the repaired tendon so that it may absorb the growth factors from the previously created marrow vents (Fig 3).

### Rotator Cuff Repair

A standard double-row rotator cuff repair may now be performed. This may be performed by passing the medial-row sutures in a horizontal mattress fashion from the articular side to the bursal side of the torn rotator cuff tendon. Final adjustment of the wick with a grasper to lie flat against the humeral head may be performed, and the medial-row knots may be tied (Fig 1C). If significant residual wick is protruding from beneath the repair, a biter (DePuy Mitek) may be used to remove the excess but leaving some residual showing laterally is also acceptable. Lateral-row anchors are then placed incorporating the medial-row anchors. Pearls and pitfalls of the described technique are presented in Table 1, and advantages and disadvantages are listed in Table 2.

**Table 1:** Pearls and Pitfalls

Pearls
Slide the scaffold over the sutures from a medial-row anchor prior to passing the sutures through the cuff.
Ensure the graft lies flat against the humeral head prior to tying the medial-row knots.

Pitfalls
Avoid passing the suture through the graft near the edges to prevent graft bunching.
Take care when passing the graft through the cannula owing to graft fragility.

**Table 2:** Advantages and Disadvantages

Advantages
Easy to implant
Scaffold resorption over period of 3-4 mo
Improved rotator cuff strength at 3 mo in animal models
Low retear rates in prospective studies

Disadvantages
Cost
Not yet compared with rotator cuff repair alone in RCT

RCT, randomized controlled trial.

## Discussion

Despite significant advances in arthroscopic rotator cuff repair over the past few decades, there is still a high rate of repair failure.^2^, ^3^, ^4^ Several strategies to decrease the rate of failure have been proposed, ranging from local adjuvants such as platelet-rich plasma and bone marrow aspirate to patch augmentation. Most augmentations are designed to act as a scaffold both to provide immediate supplemental strength to the construct and to augment the local healing response. Acellular dermal patches augmented with platelet-rich plasma and bone marrow aspirate have been used as an augmentation in the setting of revision massive rotator cuff repair; however, a significant failure rate of 59%, which may be expected in this population, was noted.^10^ Scaffolds created from reconstituted bovine collagen have shown promising results in patients undergoing treatment of partial- and full-thickness rotator cuff tears, with greater than 80% and 70% of patients, respectively, showing meaningful clinical improvement.^9^ Several complications were observed in the postoperative period, however, including a case of partial graft migration leading to stiffness. The use of these augmentations may also be technically challenging as well as time-consuming, and when placed on top of the tendon, they have no impact on the healing at the bone-tendon interface, where failure to heal likely occurs.

**Fig 4:**
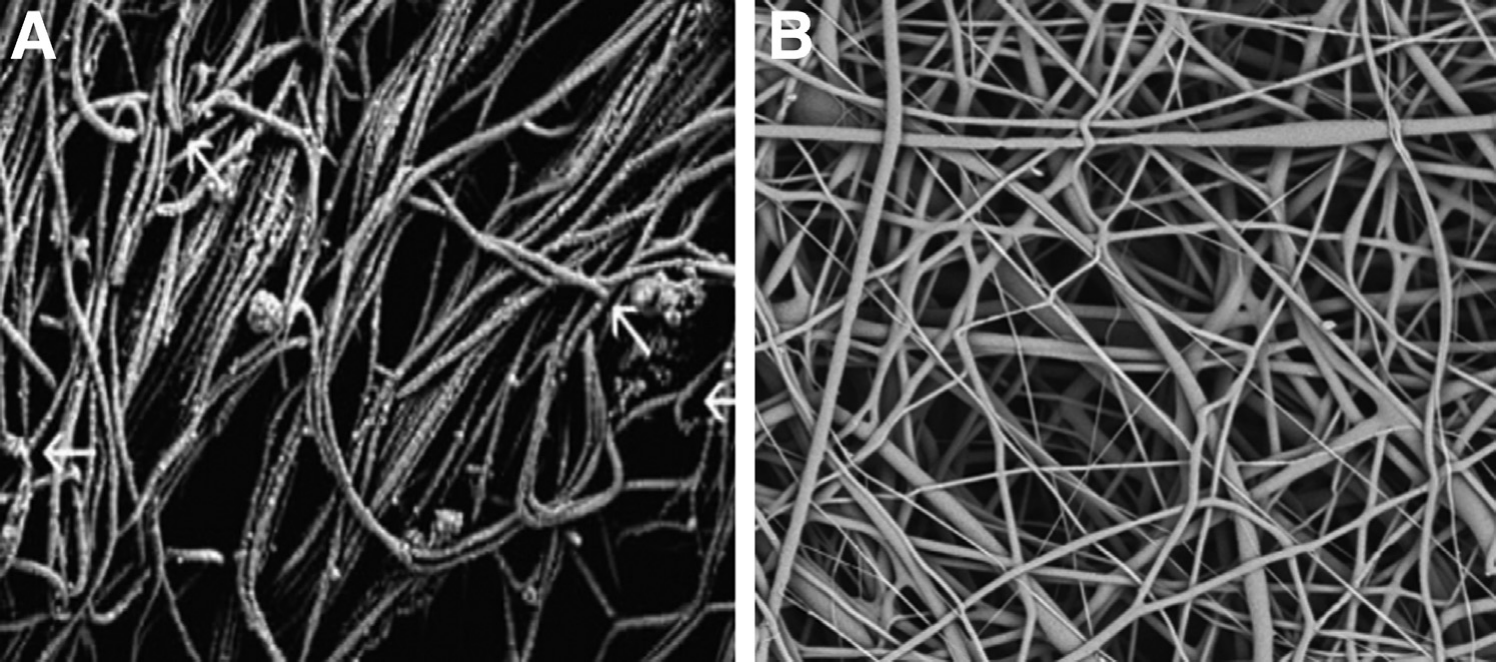
Scanning electron micrographs of native tendon extracellular matrix (ECM) fibers (arrows) (A) and Rotium wick (B), whose fibers act as a scaffold for cellular proliferation and ingrowth to allow for regeneration of tendon and Sharpey fibers. Original magnification ×1400 (Reprinted from Atreon Orthopedics with permission.)

The Rotium wick interpositional scaffold is easy to use, requiring minimal additional operative time for placement, and does not require any special instrumentation. It is composed of a polyglycolic acid (PGA)–poly-L-lactic-co-ε-caprolactone (PLCL) polymer that mimics the native tendon’s extracellular matrix structure (Fig 4).^8^ The structure of the nanofibers in conjunction with the scaffold’s microporous nature is thought to promote a healing response, and the scaffold is intended to resorb over a period of 3 to 4 months. In a sheep model, the use of an interpositional nanofiber scaffold in conjunction with a double-row repair for an acute rotator cuff tear increased the ultimate force to failure and increased the strength of the repair.^8^ At 12 weeks, the organization of the tendon fibers at the insertion point was more like the native anatomy than was found in sheep treated without a scaffold.

A recently published study reported on a retrospective cohort of patients undergoing arthroscopic rotator cuff repair with an interpositional scaffold.^7^ The study showed a 9% rate of failure, primarily in the setting of large or massive tear patterns, which are known to be associated with increased rates of failure.^2^^,^^3^ When small- to medium-sized tears, which comprised most of this cohort, were segregated, a 97% healing rate was noted based on postoperative magnetic resonance imaging analysis. In addition, scaffold reabsorption was observed on magnetic resonance imaging in all patients at a minimum of 6 months postoperatively, with no adverse events noted. Although this small retrospective study has shown promising results regarding both efficacy and safety, a prospective multicenter comparative study is currently under way.

Advantages of the described technique include the placement at the bone-tendon interface, putting the polymer directly where healing is to occur to better promote cell migration and adhesion, as opposed to lying on the bursal side of the rotator cuff as occurs with many scaffolds on the market. The use of a synthetic scaffold also may decrease the risk of an inflammatory response to a biological scaffold. Moreover, the lack of special instrumentation decreases potential costs, and the procedure itself adds minimal time to an operation. However, it remains to be seen if the benefits of prevented symptomatic retears outweigh the cost of the implant itself. Other limitations include the lack of data in patients with larger tears, although this will be further evaluated in an in-progress randomized controlled trial. There is always a concern of graft migration when a foreign body is introduced; however, this has not yet been seen in the available literature.

The interpositional nanofiber scaffold is easy to use and has shown promising results for use in rotator cuff repairs in human and animal models, and it can be a useful tool for the shoulder surgeon.

## Supplementary Data

ICMJE author disclosure forms

Video 1 Rotator cuff repair with interpositional nanofiber scaffold augmentation. Arthroscopy is performed on the right shoulder with the patient in the beach-chair position. The rotator cuff footprint on the greater tuberosity is prepared, and medial-row anchors are placed. The nanofiber scaffold is passed onto the field. A bird-beak device is placed through the center of the scaffold and used to shuttle all sutures through the center of the scaffold. The scaffold is gently slid down the sutures through the lateral cannula and laid between the rotator cuff and greater tuberosity footprint. Sutures are then passed through the cuff in standard fashion. The medial-row sutures may be tied down (as shown in this case) or placed into the lateral-row anchor.
